# Use of Systemic Glucocorticoids and Risk of Prostate Adenocarcinoma: Evidence From a Danish Population‐Based Case–Control Study

**DOI:** 10.1002/cam4.71110

**Published:** 2025-07-30

**Authors:** Elea Olivier, Blánaid Hicks, Morten Olesen, Agnès Fournier, Gianluca Severi, Anton Pottegård, Manon Cairat

**Affiliations:** ^1^ Université Paris‐Saclay UVSQ, Inserm, Gustave Roussy, CESP Villejuif France; ^2^ Clinical Pharmacology and Pharmacy, Department of Public Health University of Southern Denmark Odense Denmark; ^3^ Centre for Public Health Queen's University Belfast Belfast UK; ^4^ Department of Statistics, Computer Science and Applications “G. Parenti” University of Florence Florence Italy; ^5^ Université de Bordeaux INSERM, BPH, Team AHeaD Bordeaux France

**Keywords:** Danish registries, glucocorticoids, pharmacoepidemiology, prostate cancer

## Abstract

**Background:**

Glucocorticoids may promote prostate cancer by reducing apoptosis and the immune response, or prevent it by reducing inflammation, inhibiting androgens, and limiting cell proliferation. However, epidemiological evidence is limited. Thus, this study aimed to assess the association between systemic glucocorticoids and prostate cancer risk within the Danish registries.

**Methods:**

A nationwide case–control study was conducted using Danish healthcare registries. Men with a primary prostate adenocarcinoma diagnosis between 2001 and 2018 were identified as cases (*n* = 56,575). For each case, 10 controls were randomly selected from the general population, matched on age and calendar time. Exposure to systemic glucocorticoid was identified via the national prescription registry from 1995 onwards. Ever users of systemic glucocorticoids were defined as at least 2 filled prescriptions, and long‐term use as filled prescriptions equivalent to ≥ 1000 defined daily doses (DDDs). Conditional logistic regressions were performed to calculate odds ratios (ORs) and 95% confidence intervals for the association between systemic glucocorticoid use and prostate cancer risk.

**Results:**

Twelve percent of men had ever been exposed to systemic glucocorticoids. No association was observed between ever and long‐term use of systemic glucocorticoids and prostate cancer risk [OR = 1.03 (1.00–1.06) and OR = 1.02 (0.92–1.14), respectively], compared with never use. However, an inverse association was observed with the highest use category (> 1500 DDDs) [OR = 0.86 (0.74–0.99)], though without evidence of a dose–response relationship [OR per 500 DDDs = 0.98 (0.95–1.01), *p* = 0.18]. Associations did not differ by prostate cancer stage.

**Conclusion:**

This large nationwide nested case–control study suggested no evidence of a higher risk of prostate adenocarcinoma associated with systemic glucocorticoid use.

## Graphical abstract

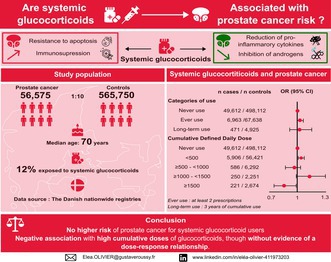

**Abbreviations**
CI: confidence interval
DDD: defined daily dose
IQR: InterQuartile Range
NA: not applicable
OR: odds ratio
RR: Relative risk

## 1 Introduction

Since their introduction in 1949 for rheumatoid arthritis treatment [1], systemic glucocorticoids have been widely used to manage various chronic conditions, including allergies, rheumatic disorders, respiratory, autoimmune, and inflammatory diseases [2, 3]. These drugs are known to promote insulin resistance, metabolic dysfunctions, and immunosuppression [3, 4, 5], which may contribute to a higher risk of several cancers, including prostate cancer [6, 7, 8]. The potential impact of systemic glucocorticoids on prostate cancer risk remains unclear. Systemic glucocorticoids may influence the risk of prostate cancer in opposite ways. They may promote tumor initiation or metastasis by reducing apoptosis and suppressing immune function [4, 9], but they may also play a protective role by reducing pro‐inflammatory cytokines, inhibiting androgens, and limiting cancer cell proliferation [10, 11, 12]. Yet, limited studies on prostate cancer have been conducted. Only three studies have examined the association between systemic glucocorticoid use and prostate cancer; all reported a higher risk of prostate cancer among systemic glucocorticoid users compared to non‐users [13, 14, 15]. However, the study from the Prostate Cancer Database Sweden [15] found no dose–response relationship and did not account for latency or methods to limit protopathic bias [16]. In the Melbourne Collaborative Cohort Study [14], the authors did not assess the cumulative exposure ‐ a critical limitation when evaluating drug‐related risks [17]. Finally, the study using the SEER‐Medicare database had notable limitations, including a restricted exposure window of 3 years, no consideration of latency periods, and a study population limited to individuals over 68 years old [13].

Thus, we used the nationwide Danish registries to investigate the associations between use of systemic glucocorticoids and prostate cancer risk.

## 2 Materials and Methods

We conducted a nested case–control study using data from Danish nationwide registries.

### 2.1 Nationwide Registry Sources

Data from the six following nationwide registry sources were used: the Danish Cancer Registry [18], the National Prescription Registry [19], the National Patient Registry [20], Registers in Statistics Denmark for educational level [21], the Danish Pathology Register [22], and the Civil Registration System [23, 24]. A description of the registries is detailed in Appendix S1.

In Denmark, the Danish National Health Service funds almost all medical care, allowing comprehensive population‐based register linkage studies that cover all residents of the country [25]. Data sources are linked by a unique personal identification number allocated to all residents since 1968 [24]. All linkages are performed by Statistics Denmark, a government agency responsible for collecting and processing data for various statistical and scientific purposes.

### 2.2 Selection of Prostate Cancer Cases and Population Controls

Prostate adenocarcinoma cases were retrieved from the Danish Cancer Registry. Cancer diagnosis codes are detailed in Table S3. All men with a histologically verified diagnosis of primary prostate adenocarcinoma between January 1st 2001 and December 31st 2018, were defined as cases. The diagnosis date was defined as the index date. For each case, we selected 10 controls from the Danish male population, matched by exact birth year and calendar time. Controls were chosen through risk set sampling and assigned the same index date as their corresponding case. Since individuals could be selected as controls before they became cases, the resulting odds ratios (ORs) provide unbiased estimates of the incidence rate ratios that would be obtained in a cohort study based on the same source population [26]. We excluded cases and controls who:

1. were either younger than 18 years or older than 85 years at the index date,
2. had a diagnosis of any other cancer (except non‐melanoma skin cancer) before the index date,
3. resided outside Denmark at any point prior to the index date,
4. had at least one prescription of systemic glucocorticoids between January 1st, 1995 and December 31st, 1995, to exclude those likely to have started treatment before prescription data became available, or,
5. died before or on the index date (prostate cancer recorded post mortem).

### 2.3 Exposure to Systemic Glucocorticoids

We retrieved all prescriptions of systemic glucocorticoids from January 1st, 1996, onward. Drug exposure codes are detailed in Table S3. To allow for a minimum latency period and minimize detection and protopathic biases, prescriptions filled in the year preceding the index date were excluded from all analyses [16]. Ever users were defined as men with at least two prescriptions between January 1st, 1996 and 1 year before the index date, while those with 0–1 prescription were classified as never users (reference category). Exposure was further categorized based on cumulative defined daily doses (DDDs), with long‐term use defined as filled prescriptions equivalent to ≥ 1000 DDDs. In addition, we conducted separate analyses for the 5 most frequently prescribed glucocorticoids, including prednisolone, betamethasone, prednisone, methylprednisolone, and hydrocortisone.

### 2.4 Covariates

Potential confounders were selected a priori based on existing literature and data availability in the registries. Prescriptions of drugs suspected to modify prostate cancer risk and likely to be associated with the use of systemic glucocorticoids were identified from the Prescription Registry. This includes immunosuppressants, non‐steroidal anti‐inflammatory drugs, proton pump inhibitors, statins, low‐dose aspirin, and selective serotonin reuptake inhibitors. Men with at least two prescriptions of the drug of interest from 1995 to 1 year prior to the index date were defined as ever users.

Diagnoses of comorbidities were identified from the Danish National Patient Registry. They were defined as a primary or secondary discharge, outpatient diagnosis, or by related medications. We considered conditions requiring glucocorticoid use, including chronic obstructive pulmonary disease, asthma, rheumatoid arthritis, polymyalgia rheumatica/giant cell arthritis, psoriasis arthritis, ankylosing spondylitis, Crohn's disease, ulcerative colitis, renal diseases, multiple sclerosis, and adrenal insufficiency. The Charlson comorbidity index score was categorized as follows: 0 (low), 1–2 (medium), or ≥ 3 (high), based on the prevalence of 19 chronic conditions [27, 28]. Comorbidities recorded within 1 year before the index date were also excluded. Information on educational level was retrieved from the registries at Statistics Denmark and the Civil Registration System and used as a crude measure of socioeconomic status (basic, medium, higher or unknown). Covariate codes are listed in Table S3.

### 2.5 Statistical Analyses

The frequency and proportion of cases and controls were calculated within categories of exposure and covariates. Conditional logistic regressions were used to estimate ORs and 95% confidence intervals for the association between systemic glucocorticoid use and prostate cancer risk. Analyses were stratified by predefined categories of cumulative doses of systemic glucocorticoids (< 500, 500–999, 1000–1499, ≥ 1500 DDDs) to explore potential dose–response associations. In all analyses, never users (defined as having < 2 prescriptions of systemic glucocorticoids) served as the reference category. In analyses of individual glucocorticoids (i.e., prednisolone, betamethasone, prednisone, methylprednisolone and hydrocortisone), the reference class was men who had never used any systemic glucocorticoids. Models were adjusted for chronic obstructive pulmonary disease, asthma, rheumatoid arthritis, polymyalgia rheumatica/giant cell arthritis, psoriatic arthritis, ankylosing spondylitis, Crohn's disease, ulcerative colitis, renal diseases, multiple sclerosis, adrenal insufficiency, Charlson comorbidity index score, educational level, ever use of immunosuppressants, non‐steroidal anti‐inflammatory drugs, proton pump inhibitors, statins, low‐dose aspirin, and selective serotonin reuptake inhibitors.

We conducted various subgroup and sensitivity analyses. First, we explored the association between systemic glucocorticoids and prostate cancer risk, stratified by clinical stage (localized, non‐localized and unknown). Then, we performed stratified analyses based on age at index date (< 55, 55–69 and ≥ 70). We also repeated the main analyses by varying the minimum latency period, ranging from 0 to 5 years (in one‐year increments). Lastly, we restricted our analyses to men diagnosed with inflammatory bowel diseases (Crohn's disease or ulcerative colitis) and those diagnosed with rheumatoid arthritis to address indication bias. All statistical analyses were conducted using STATA version 18.1.

## 3 Results

### 3.1 Patient Characteristics

**FIGURE 1:**
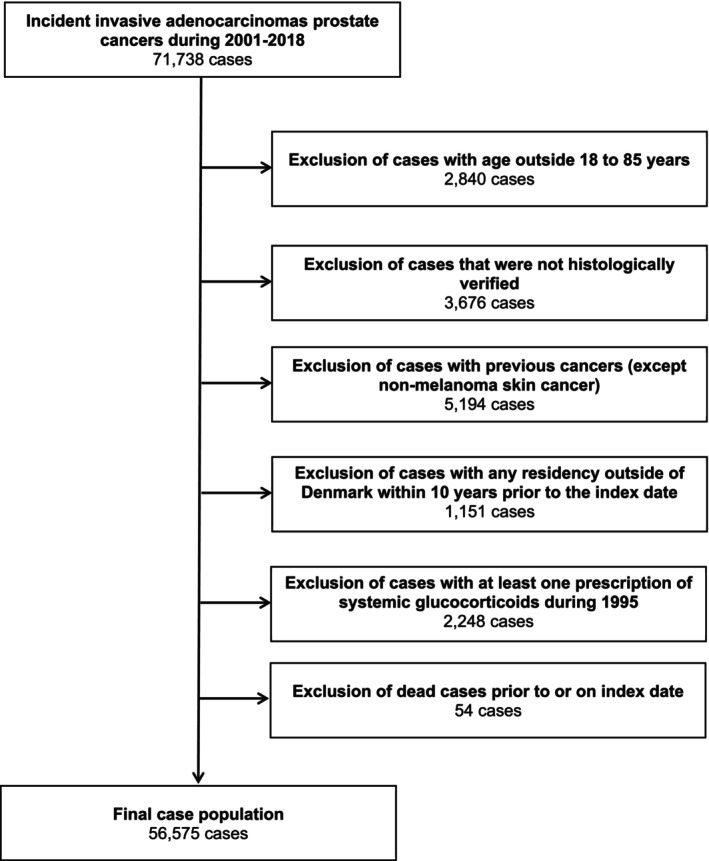
Flow‐chart of the selection of cases.

The study population included 56,575 icident cases of prostate adenocarcinoma and 565,750 controls (Figure 1). 27,278 (48%) were localized, 7539 (13%) non‐localized, and 21,758 (38%) unknown stages. Table 1 presents the study population characteristics. The median age at index date was 70 years (interquartile range, 64–75). Differences in characteristics at index date between cases and controls were generally minor, with the exception of slightly higher use of non‐steroidal anti‐inflammatory drugs and longer education among cases compared to controls. At the index date, 12% of men (for both cases and controls) had filled at least two prescriptions for systemic glucocorticoids, while less than 1% were classified as long‐term users.

**TABLE 1:** Characteristics of prostate cancer cases and matched controls.

	Cases, *n* = 56,575	Controls, *n* = 565,750
Age, median (IQR, years)	70 (64–75)	70 (64–75)
Prostate cancer stage		
Localized	27,278 (48%)	NA
Non localized	7539 (13%)	NA
Unknown	21,758 (38%)	NA
Use of systemic glucocorticoids		
Never	49,612 (88%)	498,112 (88%)
Ever^a^	6963 (12%)	67,638 (12%)
Long‐term^b^	471 (0.83%)	4925 (0.87%)
Cumulative DDDs, median (IQR)	100 (45–279)	100 (50–300)
Ever use of other drugs^a^		
Immunosuppressants	727 (1.3%)	7524 (1.3%)
Nonsteroidal anti‐inflammatory drugs	33,269 (59%)	317,387 (56%)
Proton pump inhibitors	3439 (6.1%)	35,321 (6.2%)
Statins	15,851 (28%)	161,137 (28%)
Low dose aspirin	15,603 (28%)	162,113 (29%)
Selective serotonin reuptake inhibitors	5593 (9.9%)	59,358 (10%)
Comorbidities		
Chronic obstructive pulmonary disease	9024 (16%)	90,191 (16%)
Asthma	1088 (1.9%)	10,030 (1.8%)
Rheumatoid arthritis	374 (0.66%)	4384 (0.77%)
Polymyalgia rheumatica/Giant cell arthritis	381 (0.67%)	3638 (0.64%)
Psoriatic arthritis	115 (0.20%)	1151 (0.20%)
Ankylosing spondylitis	96 (0.17%)	870 (0.15%)
Crohn's disease	119 (0.21%)	1264 (0.22%)
Ulcerative colitis	384 (0.68%)	3991 (0.71%)
Renal diseases	1035 (1.8%)	13,079 (2.3%)
Multiple sclerosis	88 (0.16%)	1118 (0.20%)
Adrenal insufficiency	35 (0.06%)	266 (0.05%)
Charlson Comorbidity Index		
None (Score = 0)	39,187 (69%)	377,178 (67%)
Low (Score = 1)	9944 (18%)	101,592 (18%)
Medium (Score = 2)	4166 (7.4%)	44,270 (7.8%)
High (Score ≥ 3)	3278 (5.8%)	42,710 (7.5%)
Highest achieved education		
Short (7–10 years)	17,151 (30%)	189,539 (34%)
Medium (11–12 years)	24,329 (43%)	240,285 (42%)
Long (≥ 13 years)	13,538 (24%)	118,925 (21%)
Missing or unknown	1557 (2.8%)	17,001 (3.0%)

Abbreviations: DDD, defined daily dose; IQR, InterQuartile Range; NA, not applicable.

^a^ Ever use was considered when filled ≥ 2 prescriptions more than 1 year prior to the index date.

^b^ Long‐term use was considered when filled prescriptions more than 1 year prior to the index date were equivalent to ≥ 1,000 DDDs.

### 3.2 Main Association Analyses

**FIGURE 2:**
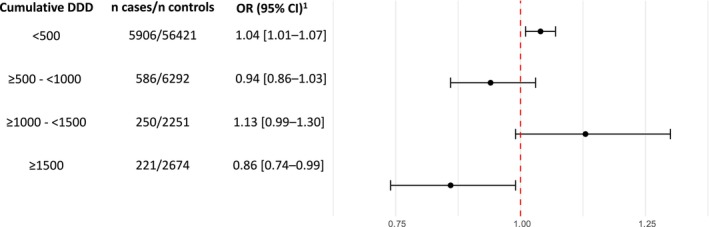
Associations of glucocorticoids use with prostate cancer risk, compared to never use, by cumulative dose. CI, confidence interval; DDD, defined daily dose; OR, odd ratio. ^1^OR adjusted for age, calendar time (by risk‐set sampling and the conditional analysis), chronic obstructive pulmonary disease, asthma, rheumatoid arthritis, polymyalgia rheumatica/giant cell arthritis, psoriasis arthritis, ankylosing spondylitis, Crohn's disease, ulcerative colitis, renal diseases, multiple sclerosis, and adrenal insufficiency, Charlson comorbidity index score, educational level, immunosuppressants, non‐steroidal anti‐inflammatory drugs, proton pump inhibitors, statins, low‐dose aspirin, and selective serotonin reuptake inhibitors.

The associations between systemic glucocorticoid use and prostate cancer risk are shown in Table 2. Compared to never users, neither ever use nor long‐term use of systemic glucocorticoids was associated with a higher risk of prostate cancer [OR_ever use_ = 1.03 (1.00–1.06) and OR_long‐term use_ = 1.02 (0.92–1.14)]. We observed a slight positive association between prostate cancer risk and exposure to low cumulative doses of systemic glucocorticoids (< 500 DDDs), compared to non‐users [OR = 1.04 (1.01–1.07)]. In contrast, a slight inverse association was observed in the highest cumulative dose category of systemic glucocorticoids (> 1500 DDDs) [OR = 0.86 (0.74–0.99)]. However, there was no clear evidence of a dose–response relationship [OR_per 500 DDDs_ = 0.98 (0.95–1.01), *p =* 0.18]. These findings are visually presented in Figure 2. Analyses of individual glucocorticoids showed no associations, and generally similar estimates associated with long‐term use were observed [OR_methylprednisolone_ = 0.67 (0.27–1.67), OR_prednisone_ = 1.00 (0.73–1.38), OR_prednisolone_ = 1.03 (0.91–1.17), OR_hydrocortisone_ = 1.06 (0.43–2.63)].

**TABLE 2:** Associations between systemic glucocorticoid use and prostate cancer risk, overall and by type of glucocorticoids.

	*n*	*n*	OR (95% CI)^a^	OR (95% CI)^b^
	Cases	Controls		
All systemic glucocorticoids				
Use categories				
Never use	49,612	498,112	1.00 (ref.)	1.00 (ref.)
Ever use	6963	67,638	1.03 (1.01–1.06)	1.03 (1.00–1.06)
Long‐term use	471	4925	0.95 (0.86–1.05)	1.02 (0.92–1.14)
Type of glucocorticoids^c^				
Betamethasone				
Ever use	2111	19,499	1.09 (1.04–1.14)	1.07 (1.02–1.12)
Long‐term use	(*n* < 5)	49	(−)	(−)
Methylprednisolone				
Ever use	876	8356	1.05 (0.97–1.12)	1.02 (0.95–1.10)
Long‐term use	5	74	0.64 (0.26–1.58)	0.67 (0.27–1.67)
Prednisone				
Ever use	515	4783	1.07 (0.98–1.18)	1.08 (0.98–1.19)
Long‐term use	45	488	0.92 (0.67–1.25)	1.00 (0.73–1.38)
Prednisolone				
Ever use	3348	34,054	0.98 (0.95–1.02)	1.00 (0.95–1.04)
Long‐term use	376	3978	0.94 (0.84–1.05)	1.03 (0.91–1.17)
Hydrocortisone				
Ever use	17	137	1.21 (0.73–2.02)	1.07 (0.59–1.93)
Long‐term use	7	53	1.26 (0.57–2.79)	1.06 (0.43–2.63)

Abbreviations: CI, confidence interval; OR, Odds ratio.

^a^ Adjusted for age and calendar time (by risk‐set sampling and the conditional analysis).

^b^ Adjusted for age, calendar time (by risk‐set sampling and the conditional analysis), chronic obstructive pulmonary disease, asthma, rheumatoid arthritis, polymyalgia rheumatica/giant cell arthritis, psoriasis arthritis, ankylosing spondylitis, Crohn's disease, ulcerative colitis, renal diseases, multiple sclerosis, and adrenal insufficiency, Charlson comorbidity index score, educational level, immunosuppressants, non‐steroidal anti‐inflammatory drugs, proton pump inhibitors, statins, low‐dose aspirin, and selective serotonin reuptake inhibitors.

^c^ The reference class was never used of any systemic glucocorticoids.

### 3.3 Subgroup and Sensitivity Analyses

The OR for the highest cumulative dose of systemic glucocorticoids (≥ 1500 DDDs) was 0.83 (0.70–0.99) in men aged ≥ 70 years, and 0.92 (0.72–1.18) in those aged 55–69 years. By tumor stage, the ORs were 0.83 (0.67–1.03) for localized prostate cancer, and 1.00 (0.71–1.42) for non‐localized prostate cancer, 0.84 (0.67–1.07) for unknown stage. However, these subgroup differences were not statistically significant (*p*_homogeneity_ ≥ 0.07, Table 3 and tumor stage: *p*_homogeneity_ ≥ 0.1, Table 4) and may reflect random variation rather than significant heterogeneity. We observed a slightly higher risk of prostate cancer associated with ever use of systemic glucocorticoids, particularly when analyses did not account for a lag time ([OR = 1.08 (1.05–1.11)], Table S1). This association was slightly attenuated when exposure was lagged by 1 year [OR = 1.03 (1.00–1.06)]. Findings remained stable when modifying the latency period from 0 to 5 years (Table S1). Similarly, restricting the analyses to patients with a diagnosis of inflammatory bowel disease or rheumatoid arthritis yielded comparable results (Table S2).

**TABLE 3:** Associations of systemic glucocorticoid use with risk of prostate cancer, stratified by age at index date.

	Age < 55 years			Age ≥ 55 and < 70 years			Age ≥ 70 years			*p*_heterogeneity_
	*n*	*n*	OR (95% CI)^a^	*n*	*n*	OR (95% CI)^a^	*n*	*n*	OR (95% CI)^a^	
	Cases	Controls		Cases	Controls		Cases	Controls		
Use categories										
Never use	1579	15,707	1.00 (ref.)	22,900	230,999	1.00 (ref.)	25,133	251,406	1.00 (ref.)	
Ever use	114	1223	0.90 (0.73–1.11)	2731	25,311	1.07 (1.02–1.12)	4118	41,104	1.01 (0.97–1.05)	0.07
Long‐term use	5	52	1.00 (0.36–2.76)	147	1420	1.09 (0.90–1.32)	319	3453	0.98 (0.85–1.12)	0.68
Cumulative DDDs										
Never use	1579	15,707	1.00 (ref.)	22,900	230,999	1.00 (ref.)	25,133	251,406	1.00 (ref.)	
< 500	99	1104	0.87 (0.70–1.08)	2420	22,119	1.07 (1.03–1.13)	3387	33,198	1.02 (0.98–1.06)	
≥ 500–< 1000	10	67	1.48 (0.73–3.01)	164	1772	0.94 (0.79–1.11)	412	4453	0.93 (0.83–1.03)	
≥ 1000–< 1500	(*n* < 5)	24	(−)	75	603	1.28 (1.00–1.64)	172	1624	1.07 (0.91–1.26)	
≥ 1500	(*n* < 5)	28	(−)	72	817	0.92 (0.72–1.18)	147	1829	0.83 (0.70–0.99)	
OR per 500 DDDs	114	1223	0.99 (0.77–1.28)	2731	25,311	1.01 (0.96–1.06)	4118	41,104	0.97 (0.93–1.00)	
*p*‐value			0.96			0.67			0.05	

Abbreviations: CI, confidence interval; DDD, defined daily dose; OR, odds ratio.

^a^ Adjusted for age, calendar time (by risk‐set sampling and the conditional analysis), chronic obstructive pulmonary disease, asthma, rheumatoid arthritis, polymyalgia rheumatica/giant cell arthritis, psoriasis arthritis, ankylosing spondylitis, Crohn's disease, ulcerative colitis, renal diseases, multiple sclerosis, and adrenal insufficiency, Charlson comorbidity index score, educational, immunosuppressants, non‐steroidal anti‐inflammatory drugs, proton pump inhibitors, statins, low‐dose aspirin, and selective serotonin reuptake inhibitor.

**TABLE 4:** Associations of systemic glucocorticoid use with risk of prostate cancer by tumor stage.

	Localized			Non localized			Unknown			*p*_heterogeneity_
	*n*	*n*	OR (95% CI)^a^	*n*	*n*	OR (95% CI)^a^	*n*	*n*	OR (95% CI)^a^	
							Cases	Controls		
	Cases	Controls		Cases	Controls					
Use categories										
Never use	23,813	240,064	1.00 (ref.)	6633	65,726	1.00 (ref.)	19,166	192,322	1.00 (ref.)	
Ever use	3465	32,716	1.06 (1.02–1.10)	906	9664	0.97 (0.89–1.04)	2592	25,258	1.02 (0.97–1.07)	0.10
Long‐term use	214	2333	1.00 (0.85–1.18)	83	760	1.22 (0.92–1.60)	174	1832	0.98 (0.82–1.18)	0.47
Cumulative DDDs										
Never use	23,813	240,064	1.00 (ref.)	6633	65,726	1.00 (ref.)	19,166	192,322	1.00 (ref.)	
< 500	2973	27,607	1.07 (1.02–1.11)	749	7936	0.97 (0.89–1.05)	2184	20,878	1.03 (0.98–1.08)	
≥ 500–< 1000	278	2776	1.01 (0.89–1.15)	74	968	0.79 (0.62–1.01)	234	2548	0.92 (0.80–1.06)	
≥ 1000–< 1500	114	1069	1.08 (0.88–1.32)	44	345	1.31 (0.94–1.83)	92	837	1.12 (0.90–1.40)	
≥ 1500	100	1264	0.83 (0.67–1.03)	39	415	1.00 (0.71–1.42)	82	995	0.84 (0.67–1.07)	
OR per 500 DDDs	3465	32,716	0.99 (0.95–1.03)	906	9664	0.99 (0.92–1.07)	2592	25,258	0.97 (0.93–1.02)	
*p*‐value			0.53			0.82			0.23	

Abbreviations: CI, confidence interval; DDD, defined daily dose; OR, odds ratio.

^a^ Adjusted for age, calendar time (by risk‐set sampling and the conditional analysis), chronic obstructive pulmonary disease, asthma, rheumatoid arthritis, polymyalgia rheumatica/giant cell arthritis, psoriatic arthritis, ankylosing spondylitis, Crohn's disease, ulcerative colitis, renal diseases, multiple sclerosis, and adrenal insufficiency, Charlson comorbidity index score, education, immunosuppressants, non‐steroidal anti‐inflammatory drugs, proton pump inhibitors, statins, low‐dose aspirin, and selective serotonin reuptake inhibitors.

## 4 Discussion

In this large nationwide registry‐based study, we found no evidence of an increased risk of prostate cancer with systemic glucocorticoid use. Although a lower prostate cancer risk was observed in the highest category of cumulative dose of systemic glucocorticoids (> 1500 DDDs), there was no evidence of a dose–response relationship.

Our results contrast with three previous studies that suggested a positive association between glucocorticoids and prostate cancer risk [13, 14, 15]. Using the SEER‐Medicare database, Singh et al. conducted the first study specifically designed to assess this association [13]. They reported an increased risk of advanced prostate cancer with prolonged glucocorticoid use (> 2 years) [relative risk (RR) = 1.74 (1.12–2.69), *n* exposed cases = 205], with a dose–response relationship [RR = 1.08 (1.01–1.16)]. However, the absence of a lag time could potentially explain the positive association observed due to reverse causality [16, 29]. In an Australian cohort study investigating the effect of asthma and associated medications on prostate cancer risk, Severi et al. found a positive association between ever use of systemic glucocorticoids and prostate cancer risk [RR = 1.71 (1.08–2.69), n exposed cases = 19] [14], though they did not provide data on the dose–response relationship. Similarly, within a nested case–control study investigating the associations between chronic inflammatory diseases, anti‐inflammatory drugs, and prostate cancer risk, Beckmann et al. observed a positive association between systemic glucocorticoid ever use and prostate cancer risk [RR = 1.14 (1.11–1.17), *n* exposed cases = 7449], with no evidence of a dose–response relationship [15]. Consistent with previous studies, we observed a slightly higher risk of prostate cancer associated with the ever use of systemic glucocorticoids, particularly when no lag was applied. This association was slightly attenuated after the introduction of a one‐year lag. Therefore, reverse causation may be one possible explanation for the positive associations reported in previous studies that did not incorporate a lag period [13, 14, 15] ‐ that is, early symptoms of undiagnosed prostate cancer may have led to glucocorticoid prescriptions shortly before diagnosis, creating a spurious association. Moreover, the absence of a consistent dose–response pattern, the opposing directions of association at short and long cumulative exposure categories, and the sensitivity of the results to lag time, all suggest that the observed associations are unlikely to reflect a robust or clinically meaningful causal relationship. Conversely, Su et al. found no association between glucocorticoid use and prostate cancer risk in a case‐cohort study examining the association between asthma and risk of prostate cancer [30]. However, their findings should be interpreted with caution, as they did not specify the route of glucocorticoid administration, and both the main exposure (asthma) and confounding factors (including glucocorticoids) were included in a single model. This may have led to interpretation challenges and confusion between direct and total effect estimates for the covariates in the model [31].

Although previous studies suggested that glucocorticoids may increase the risk of several cancers [32, 33, 34, 35], particularly due to immunosuppression, our results did not support the evidence of an increased risk of prostate cancer with systemic glucocorticoid use. Instead, we observed a slight inverse association at the highest cumulative exposure dose. One potential explanation could be that systemic glucocorticoids exert negative feedback on the hypothalamic–pituitary axis, leading to the suppression of testicular and adrenal androgen synthesis [36, 37, 38]. Additional mechanisms could involve the inhibition of angiogenesis [39] or the suppression of inflammatory and growth factors [10, 40, 41]. However, the lack of a consistent dose–response relationship argues against a causal relationship. These results may be due to residual bias and should be interpreted with caution. One possible explanation for the inverse association observed with long‐term glucocorticoid use is selection bias. Men exposed to long‐term systemic glucocorticoids may represent a selected subgroup with a lower baseline risk ‐ for example, due to better tolerance to glucocorticoids, fewer adverse effects, or the absence of pre‐existing but undiagnosed prostate cancer at treatment initiation [42, 43, 44]. Confounding factors, such as chronic conditions common among long‐term users that may reduce cancer screening or detection, could also contribute to a spurious protective association, making it difficult to draw definitive conclusions [45].

To our knowledge, this is the largest study to date assessing the association between systemic glucocorticoid use and prostate cancer risk. A major strength of our study is the use of a high‐quality nationwide data, ensuring comprehensive cancer diagnosis across the entire Danish population while limiting the risk of selection bias [18, 46]. Furthermore, the use of the Danish Prescription Registry provided complete and reliable data on systemic glucocorticoid prescriptions, reducing the risk of differential recall bias between cases and controls, while ensuring accurate and precise information on exposure. The large size and long follow‐up ‐ up to 17 years, with prescription data spanning a period of up to 23 years—allowed detailed analysis by cancer stage and cumulative dose of glucocorticoid use. We were also able to adjust our models for socioeconomic parameters and use of other drugs. In addition, we were able to leverage detailed hospital diagnosis data on comorbidities to address indication bias. However, some limitations should be noted. First, prescription data may introduce misclassification, particularly with regard to hospital treatment and non‐adherence, although any misclassification is very likely to be non‐differential, that is, independent of case status, leading to conservative bias. However, it is likely that this may be less of a concern for long‐term users of glucocorticoids. Second, we were unable to condition our analyses for certain risk factors for prostate cancer, including ethnicity, obesity, and physical activity [47]. These factors might also be associated with use of glucocorticoids either positively or inversely, introducing a risk of residual confounding. For example, obesity and lack of physical activity have been associated with a higher risk of several chronic inflammatory diseases [48, 49, 50], many of which are commonly treated with systemic glucocorticoids. Since these factors are related to glucocorticoid use mainly through their association with chronic inflammatory diseases, we believe that adjusting for underlying inflammatory diseases, as done in our study, can at least partially account for the confounding due to these factors. In terms of ethnicity, prostate cancer is more prevalent in African Americans and Caribbean people of African descent, while it is less common in Asian Americans, Hispanics, and Latinos than in non‐Hispanic whites [51]. Although the association between ethnicity and glucocorticoid use is not well established, differences in inflammatory disease burden and access to care may influence both exposure and outcome. However, given the relatively ethnically homogeneous population in Denmark, the risk of confounding by ethnicity in our study is probably limited [52]. In addition, a limitation of this study is the lack of data on the Gleason score, which is a key indicator of tumor aggressiveness in prostate cancer [53]. Finally, we were unable to account for medical follow‐up in our analyses, which may have led to an underestimation of the association.

## 5 Conclusion

In summary, this large nationwide nested case–control study found no evidence of a positive association between systemic glucocorticoid use and the risk of prostate adenocarcinoma. While a slight inverse association was observed at the highest cumulative exposure level, the lack of a dose–response pattern suggests caution in interpretation.

## Author Contributions

**Elea Olivier:** writing – original draft, writing – review and editing, visualization, validation, methodology, resources, project administration, funding acquisition, conceptualization, investigation. **Blánaid Hicks:** writing – review and editing, data curation, validation, methodology. **Morten Olesen:** writing – review and editing, methodology, software, formal analysis, data curation, validation. **Agnès Fournier:** writing – review and editing, validation, methodology. **Gianluca Severi:** writing – review and editing, supervision, validation, methodology. **Anton Pottegård:** writing – review and editing, validation, data curation, methodology. **Manon Cairat:** supervision, writing – original draft, conceptualization, funding acquisition, investigation, validation, visualization, writing – review and editing, methodology, project administration, resources.

## Ethics Statement

No ethical approval was required for this study. The study was registered in the University of Southern Denmark's research repository, and necessary permissions for data extraction were obtained from Statistics Denmark.

## Conflicts of Interest

A.P. has participated in research projects funded by Alcon, Almirall, Astellas, AstraZeneca, Boehringer‐Ingelheim, Novo Nordisk, Servier, and LEO Pharma, all of which were regulator‐mandated phase IV‐studies. Additionally, A.P. has received an unrestricted research grant from Novo Nordisk. None of these have any relation to the current study. The remaining authors declare have no conflicts of interest.

## Supporting information


Data S1.

## Data Availability

This study relies on anonymized registry data located on a secure platform at Statistics Denmark. Access to these data requires the relevant data permits. Further details can be provided upon request by the corresponding author. Due to Danish legal regulations, data from the Danish national health registers cannot be shared. However, any researcher can apply for access through the Danish health authorities.

## References

[1] D. Le Vay and G. Loxton , “Deoxycortone Acetate and Ascorbic Acid in the Treatment of Rheumatoid Arthritis,” Lancet (London, England) 2, no. 6590 (1949): 1134. 15396965 10.1016/s0140-6736(49)91149-6

[2] C. M. Spies , C. Strehl , M. C. van der Goes , J. W. J. Bijlsma , and F. Buttgereit , “Glucocorticoids,” Best Practice & Research. Clinical Rheumatology 25, no. 6 (2011): 891–900. 22265268 10.1016/j.berh.2011.11.002

[3] O. Bereshchenko , S. Bruscoli , and C. Riccardi , “Glucocorticoids, Sex Hormones, and Immunity,” Frontiers in Immunology 9 (2018): 1332. 29946321 10.3389/fimmu.2018.01332 PMC6006719

[4] D. W. Cain and J. A. Cidlowski , “Immune Regulation by Glucocorticoids,” Nature Reviews Immunology 17, no. 4 (2017): 233–247. 10.1038/nri.2017.1 PMC9761406 28192415

[5] R. Dumbell , O. Matveeva , and H. Oster , “Circadian Clocks, Stress, and Immunity,” Frontiers in Endocrinology 7 (2016): 37. 27199894 10.3389/fendo.2016.00037 PMC4852176

[6] K. Esposito , P. Chiodini , A. Colao , A. Lenzi , and D. Giugliano , “Metabolic Syndrome and Risk of Cancer: A Systematic Review and Meta‐Analysis,” Diabetes Care 35, no. 11 (2012): 2402–2411. 23093685 10.2337/dc12-0336 PMC3476894

[7] A. V. Hernandez , V. Pasupuleti , V. A. Benites‐Zapata , P. Thota , A. Deshpande , and F. R. Perez‐Lopez , “Insulin Resistance and Endometrial Cancer Risk: A Systematic Review and Meta‐Analysis,” European Journal of Cancer 51, no. 18 (2015): 2747–2758. 26597445 10.1016/j.ejca.2015.08.031

[8] A. V. Hernandez , M. Guarnizo , Y. Miranda , et al., “Association Between Insulin Resistance and Breast Carcinoma: A Systematic Review and Meta‐Analysis,” PLoS One 9, no. 6 (2014): e99317. 24911052 10.1371/journal.pone.0099317 PMC4049776

[9] I. Herr and J. Pfitzenmaier , “Glucocorticoid Use in Prostate Cancer and Other Solid Tumours: Implications for Effectiveness of Cytotoxic Treatment and Metastases,” Lancet Oncology 7, no. 5 (2006): 425–430. 16648047 10.1016/S1470-2045(06)70694-5

[10] K. S. Sfanos , H. A. Hempel , and A. M. De Marzo , “The Role of Inflammation in Prostate Cancer,” Advances in Experimental Medicine and Biology 816 (2014): 153–181. 24818723 10.1007/978-3-0348-0837-8_7

[11] M. Puhr , J. Hoefer , A. Eigentler , et al., “The Glucocorticoid Receptor Is a Key Player for Prostate Cancer Cell Survival and a Target for Improved Antiandrogen Therapy,” Clinical Cancer Research: An Official Journal of the American Association for Cancer Research 24, no. 4 (2018): 927–938. 29158269 10.1158/1078-0432.CCR-17-0989

[12] B. Montgomery , H. H. Cheng , J. Drechsler , and E. A. Mostaghel , “Glucocorticoids and Prostate Cancer Treatment: Friend or Foe?,” Asian Journal of Andrology 16, no. 3 (2014): 354–358. 24625881 10.4103/1008-682X.125392 PMC4023359

[13] Z. Singh , S. K. Holt , J. L. Gore , Y. A. Nyame , J. L. Wright , and G. R. Schade , “Chronic Glucocorticoid Use and Risk for Advanced Prostate Cancer at Presentation: A SEER‐Medicare Cohort Study,” Clinical Genitourinary Cancer 22, no. 2 (2024): 68–73. 37806926 10.1016/j.clgc.2023.08.007

[14] G. Severi , L. Baglietto , D. C. Muller , et al., “Asthma, Asthma Medications, and Prostate Cancer Risk,” Cancer Epidemiology, Biomarkers & Prevention: A Publication of the American Association for Cancer Research, Cosponsored by the American Society of Preventive Oncology 19, no. 9 (2010): 2318–2324. 10.1158/1055-9965.EPI-10-0381 20671137

[15] K. Beckmann , B. Russell , D. Josephs , et al., “Chronic Inflammatory Diseases, Anti‐Inflammatory Medications and Risk of Prostate Cancer: A Population‐Based Case‐Control Study,” BMC Cancer 19, no. 1 (2019): 612. 31226970 10.1186/s12885-019-5846-3 PMC6588859

[16] B. Hicks , J. A. Kaye , L. Azoulay , K. B. Kristensen , L. A. Habel , and A. Pottegård , “The Application of Lag Times in Cancer Pharmacoepidemiology: A Narrative Review,” Annals of Epidemiology 84 (2023): 25–32. 37169040 10.1016/j.annepidem.2023.05.004

[17] A. Pottegård , S. Friis , T. Stürmer , J. Hallas , and S. Bahmanyar , “Considerations for Pharmacoepidemiological Studies of Drug‐Cancer Associations,” Basic and Clinical Pharmacology and Toxicology 122, no. 5 (2018): 451–459. 29265740 10.1111/bcpt.12946 PMC7025319

[18] M. L. Gjerstorff , “The Danish Cancer Registry,” Scandinavian Journal of Public Health 39, no. 7 Suppl (2011): 42–45. 21775350 10.1177/1403494810393562

[19] H. Wallach Kildemoes , H. Toft Sørensen , and J. Hallas , “The Danish National Prescription Registry,” Scandinavian Journal of Public Health 39, no. 7_suppl (2011): 38–41. 21775349 10.1177/1403494810394717

[20] M. Schmidt , S. A. J. Schmidt , J. L. Sandegaard , V. Ehrenstein , L. Pedersen , and H. T. Sørensen , “The Danish National Patient Registry: A Review of Content, Data Quality, and Research Potential,” Clinical Epidemiology 7 (2015): 449. 26604824 10.2147/CLEP.S91125 PMC4655913

[21] V. M. Jensen and A. W. Rasmussen , “Danish Education Registers,” Scandinavian Journal of Public Health 39, no. 7 Suppl (2011): 91–94. 21775362 10.1177/1403494810394715

[22] B. Bjerregaard and O. B. Larsen , “The Danish Pathology Register,” Scandinavian Journal of Public Health 39, no. 7 Suppl (2011): 72–74. 21775357 10.1177/1403494810393563

[23] M. Schmidt , L. Pedersen , and H. T. Sørensen , “The Danish Civil Registration System as a Tool in Epidemiology,” European Journal of Epidemiology 29, no. 8 (2014): 541–549. 24965263 10.1007/s10654-014-9930-3

[24] C. B. Pedersen , “The Danish Civil Registration System,” Scandinavian Journal of Public Health 39, no. 7 Suppl (2011): 22–25. 21775345 10.1177/1403494810387965

[25] L. C. Thygesen , C. Daasnes , I. Thaulow , and H. Brønnum‐Hansen , “Introduction to Danish (Nationwide) Registers on Health and Social Issues: Structure, Access, Legislation, and Archiving,” Scandinavian Journal of Public Health 39, no. 7_suppl (2011): 12–16. 21898916 10.1177/1403494811399956

[26] K. J. Rothman , S. Greenland , and T. L. Lash , eds., Modern Epidemiology, 3rd Editio ed. (Wolters Kluwer Health/Lippincott Williams & Wilkins, 2008).

[27] M. E. Charlson , P. Pompei , K. L. Ales , and C. R. MacKenzie , “A New Method of Classifying Prognostic Comorbidity in Longitudinal Studies: Development and Validation,” Journal of Chronic Diseases 40, no. 5 (1987): 373–383. 3558716 10.1016/0021-9681(87)90171-8

[28] S. K. Thygesen , C. F. Christiansen , S. Christensen , T. L. Lash , and H. T. Sørensen , “The Predictive Value of ICD‐10 Diagnostic Coding Used to Assess Charlson Comorbidity Index Conditions in the Population‐Based Danish National Registry of Patients,” BMC Medical Research Methodology 11 (2011): 83. 21619668 10.1186/1471-2288-11-83 PMC3125388

[29] A. Pottegård and J. Hallas , “New Use of Prescription Drugs Prior to a Cancer Diagnosis,” Pharmacoepidemiology and Drug Safety 26, no. 2 (2017): 223–227. 27889931 10.1002/pds.4145 PMC5299521

[30] Y. L. Su , C. L. Chou , K. M. Rau , and C. T. C. Lee , “Asthma and Risk of Prostate Cancer: A Population‐Based Case‐Cohort Study in Taiwan,” Medicine (Baltimore) 94, no. 36 (2015): e1371. 26356691 10.1097/MD.0000000000001371 PMC4616655

[31] D. Westreich and S. Greenland , “The Table 2 Fallacy: Presenting and Interpreting Confounder and Modifier Coefficients,” American Journal of Epidemiology 177, no. 4 (2013): 292–298. 23371353 10.1093/aje/kws412 PMC3626058

[32] M. Cairat , M. Al Rahmoun , M. J. Gunter , et al., “Use of Systemic Glucocorticoids and Risk of Breast Cancer in a Prospective Cohort of Postmenopausal Women,” BMC Medicine 19, no. 1 (2021): 186. 34340701 10.1186/s12916-021-02004-6 PMC8330083

[33] A. Ø. Jensen , H. F. Thomsen , M. C. Engebjerg , et al., “Use of Oral Glucocorticoids and Risk of Skin Cancer and Non‐Hodgkin's Lymphoma: A Population‐Based Case‐Control Study,” British Journal of Cancer 100, no. 1 (2009): 200–205. 19034275 10.1038/sj.bjc.6604796 PMC2634665

[34] E. B. Ostenfeld , R. Erichsen , O. Thorlacius‐Ussing , A. H. Riis , and H. T. Sørensen , “Use of Systemic Glucocorticoids and the Risk of Colorectal Cancer,” Alimentary Pharmacology & Therapeutics 37, no. 1 (2013): 146–152. 23116185 10.1111/apt.12115

[35] K. Dietrich , A. Schned , J. Fortuny , et al., “Glucocorticoid Therapy and Risk of Bladder Cancer,” British Journal of Cancer 101, no. 8 (2009): 1316–1320. 19773763 10.1038/sj.bjc.6605314 PMC2768444

[36] A. Alder , H. Burger , J. Davis , et al., “Carcinoma of Prostate: Response of Plasma Luteinizing Hormone and Testosterone to Oestrogen Therapy,” British Medical Journal 1, no. 5583 (1968): 28–30. 5636740 10.1136/bmj.1.5583.28 PMC1984910

[37] R. Tomić , B. Ljungberg , and J. E. Damber , “Hormonal Effects of High Dose Medroxyprogesterone Acetate Treatment in Males With Renal or Prostatic Adenocarcinoma,” Scandinavian Journal of Urology and Nephrology 22, no. 1 (1988): 15–18. 2968646 10.1080/00365599.1988.11690377

[38] S. Alesci , C. A. Koch , S. R. Bornstein , and K. Pacak , “Adrenal Androgens Regulation and Adrenopause,” Endocrine Regulations 35, no. 2 (2001): 95–100. 11563938

[39] A. Yano , Y. Fujii , A. Iwai , Y. Kageyama , and K. Kihara , “Glucocorticoids Suppress Tumor Angiogenesis and In Vivo Growth of Prostate Cancer Cells,” Clinical Cancer Research: An Official Journal of the American Association for Cancer Research 12, no. 10 (2006): 3003–3009. 16707595 10.1158/1078-0432.CCR-05-2085

[40] S. Vasto , G. Carruba , G. Candore , E. Italiano , D. Di Bona , and C. Caruso , “Inflammation and Prostate Cancer,” Future Oncology (London, England) 4 (2008): 637–645. 18922121 10.2217/14796694.4.5.637

[41] S. Sutcliffe and E. A. Platz , “Inflammation in the Etiology of Prostate Cancer: An Epidemiologic Perspective,” Urologic Oncology 25, no. 3 (2007): 242–249. 17483023 10.1016/j.urolonc.2006.09.014

[42] M. J. Stensrud and M. A. Hernán , “Why Test for Proportional Hazards?,” Journal of the American Medical Association 323, no. 14 (2020): 1401–1402. 32167523 10.1001/jama.2020.1267 PMC11983487

[43] M. A. Hernán , “The Hazards of Hazard Ratios,” Epidemiology (Cambridge, Mass.) 21, no. 1 (2010): 13–15. 20010207 10.1097/EDE.0b013e3181c1ea43 PMC3653612

[44] L. Deng , T. Liu , C. A. Liu , et al., “The Association of Metabolic Syndrome Score Trajectory Patterns With Risk of All Cancer Types,” Cancer 130, no. 12 (2024): 2150–2159. 38462898 10.1002/cncr.35235

[45] C. Renzi , A. Kaushal , J. Emery , et al., “Comorbid Chronic Diseases and Cancer Diagnosis: Disease‐Specific Effects and Underlying Mechanisms,” Nature Reviews Clinical Oncology 16, no. 12 (2019): 746–761. 10.1038/s41571-019-0249-6 31350467

[46] A. Pottegård , S. A. J. Schmidt , H. Wallach‐Kildemoes , H. T. Sørensen , J. Hallas , and M. Schmidt , “Data Resource Profile: The Danish National Prescription Registry,” International Journal of Epidemiology 46, no. 3 (2017): 798–798f. 27789670 10.1093/ije/dyw213 PMC5837522

[47] O. Bergengren , K. R. Pekala , K. Matsoukas , et al., “Update on Prostate Cancer Epidemiology and Risk Factors‐A Systematic Review,” European Urology 84, no. 2 (2023): 191–206. 37202314 10.1016/j.eururo.2023.04.021 PMC10851915

[48] T. Ohno , D. Aune , and A. K. Heath , “Adiposity and the Risk of Rheumatoid Arthritis: A Systematic Review and Meta‐Analysis of Cohort Studies,” Scientific Reports 10, no. 1 (2020): 16006. 32994434 10.1038/s41598-020-71676-6 PMC7524740

[49] E. Gremese , B. Tolusso , M. R. Gigante , and G. Ferraccioli , “Obesity as a Risk and Severity Factor in Rheumatic Diseases (Autoimmune Chronic Inflammatory Diseases),” Frontiers in Immunology 5 (2014): 576. 25426122 10.3389/fimmu.2014.00576 PMC4227519

[50] M. Marko and R. Pawliczak , “Obesity and Asthma: Risk, Control and Treatment,” Postepy Dermatologii I Alergologii 35, no. 6 (2018): 563–571. 30618522 10.5114/ada.2018.77607 PMC6320490

[51] L. Down , M. Barlow , S. E. R. Bailey , et al., “Association Between Patient Ethnicity and Prostate Cancer Diagnosis Following a Prostate‐Specific Antigen Test: A Cohort Study of 730,000 Men in Primary Care in the UK,” BMC Medicine 22, no. 1 (2024): 82. 38424555 10.1186/s12916-024-03283-5 PMC10905783

[52] Statistics Denmark , “Immigrants and Their Descendants,” https://www.dst.dk/en/Statistik/emner/borgere/befolkning/indvandrere‐og‐efterkommere.

[53] A. W. Partin , J. Yoo , H. B. Carter , et al., “The Use of Prostate Specific Antigen, Clinical Stage and Gleason Score to Predict Pathological Stage in Men With Localized Prostate Cancer,” Journal of Urology 150, no. 1 (1993): 110–114. 7685418 10.1016/s0022-5347(17)35410-1

