# Supplementary material for: Use of Systemic Glucocorticoids and Risk of Prostate Adenocarcinoma: Evidence From a Danish Population‐Based Case–Control Study
Source: Cancer Med. 2025 Jul 30;14(15):e71110. doi: 10.1002/cam4.71110 (PMC12308913; doi:10.1002/cam4.71110)
Supplement: Supplementary file 1 — Data S1. [file CAM4-14-e71110-s001.docx]

**ADDITIONAL FILE 1.**

**USE OF SYSTEMIC GLUCOCORTICOIDS AND RISK OF PROSTATE ADENOCARCINOMA EVIDENCE FROM A DANISH POPULATION-BASED CAS-CONTROL STUDY**

Elea Olivier, Blánaid Hicks, Morten Olesen, Agnès Fournier, Gianluca Severi, Anton Pottegård, Manon Cairat

| **Table S1** | Association between systemic glucocorticoid use and prostate cancer risk with exposure with lag periods from 0 to 5 years lag (page 2). |
| --- | --- |
| **Table S2** | Associations between systemic glucocorticoid use and prostate cancer risk restricted to men with a diagnosis of rheumatoid arthritis and to men with a diagnosis of inflammatory bowel diseases (page 3). |
| **Appendix 1** | Danish Nationwide Health Registries (pages 4-5). |
| **Table S3** | Codes and definitions (pages 6-7). |

**Table S1. Association between systemic glucocorticoid use and prostate cancer risk with exposure with lag periods from 0 to 5 years lag.**

|  | | **n cases** | | **n controls** | **OR (95% CI)^1^** |  |
| --- | --- | --- | --- | --- | --- | --- |
| **Lag 0 year** | |  | |  | |  |
| **Use categories** | |  | |  | |  |
| Never use | | 48,711 | | 491,724 | | 1.00 (ref.) |
| Ever use | | 7,864 | | 74,026 | | 1.08 (1.05-1.11) |
| Long-term use | | 555 | | 5,873 | | 1.00 (0.90-1.11) |
| **Lag 1 year** | |  | |  | |  |
| **Use categories** | |  | |  | |  |
| Never use | | 49,612 | | 498,112 | | 1.00 (ref.) |
| Ever use | | 6,963 | | 67,638 | | 1.03 (1.00-1.06) |
| Long-term use | | 471 | | 4,925 | | 1.02 (0.92-1.14) |
| **Lag 2 years** | |  | |  | |  |
| **Use categories** | |  | |  | |  |
| Never use | | 50,188 | | 504,096 | | 1.00 (ref.) |
| Ever use | | 6,387 | | 61,654 | | 1.04 (1.01-1.07) |
| Long-term use | | 383 | | 4,121 | | 0.98 (0.87-1.11) |
| **Lag 3 years** | |  | |  | |  |
| **Use categories** | |  | |  | |  |
| Never use | | 50,810 | | 509,961 | | 1.00 (ref.) |
| Ever use | | 5,765 | | 55,789 | | 1.03 (1.00-1.06) |
| Long-term use | | 331 | | 3,441 | | 1.01 (0.89-1.15) |
| **Lag 4 years** | |  | |  | |  |
| **Use categories** | |  | |  | |  |
| Never use | | 51,361 | | 515,613 | | 1.00 (ref.) |
| Ever use | | 5,214 | | 50,137 | | 1.04 (1.01-1.07) |
| Long-term use | | 270 | | 2,877 | | 0.98 (0.86-1.13) |
| **Lag 5 years** | |  | |  | |  |
| **Use categories** | |  | |  | |  |
| Never use | | 51,941 | | 521,211 | | 1.00 (ref.) |
| Ever use | | 4,634 | | 44,539 | | 1.04 (1.00-1.07) |
| Long-term use | | 221 | | 2,370 | | 0.98 (0.85-1.14) |

Abbreviations: OR, Odds ratio; CI, confidence interval; DDD, defined daily dose

^1^ Adjusted for age, calendar time (by risk-set matching and the conditional analysis), chronic obstructive pulmonary disease, asthma, rheumatoid arthritis, polymyalgia rheumatica/ giant cell arthritis, psoriatic arthritis, ankylosing spondylitis, Crohn’s disease, ulcerative colitis, renal diseases, multiple sclerosis, and adrenal insufficiency, charlson comorbidity index score, educational, immunosuppressants, non-steroidal anti-inflammatory drugs, proton pump inhibitors, statins, low-dose aspirin and selective serotonin reuptake inhibitors.

**Table S2. Associations between systemic glucocorticoid use and prostate cancer risk restricted to men with a diagnosis of rheumatoid arthritis and to men with a diagnosis of inflammatory bowel diseases**

|  | **n cases** | **N controls** | **OR (95% CI)^1^** |
| --- | --- | --- | --- |
| **Among men with rheumatoid arthritis** | | | |
| **Use categories** |  |  |  |
| Never use | 212 | 2,342 | 1.00 (ref.) |
| Ever use | 241 | 2,188 | 1.02 (0.82-1.27) |
| Long-term use | 57 | 487 | 1.14 (0.77-1.69) |
| **Cumulative DDDs** |  |  |  |
| Never use | 212 | 2,342 | 1.00 (ref.) |
| <500 | 144 | 1,293 | 1.02 (0.81-1.30) |
| ≥500 - <1000 | 40 | 408 | 0.92 (0.64-1.34) |
| ≥1000 - <1500 | 35 | 220 | 1.55 (1.02-2.35) |
| ≥1500 | 22 | 267 | 0.77 (0.47-1.25) |
| *OR per 500 DDDs* | 241 | 2,188 | 0.99 (0.90-1.09) |
| *p-value* |  |  | *0.83* |
| **Among men with inflammatory bowel diseases** | | | |
| **Use categories** |  |  |  |
| Never use | 331 | 3,388 | 1.00 (ref.) |
| Ever use | 190 | 1,822 | 0.88 (0.71-1.09) |
| Long-term use | 35 | 280 | 1.20 (0.74-1.94) |
| **Cumulative DDDs** |  |  |  |
| Never use | 114 | 1,127 | 0.89 (0.71-1.13) |
| <500 | 41 | 415 | 0.77 (0.53-1.13) |
| ≥500 - <1000 | 22 | 145 | 1.21 (0.73-2.01) |
| ≥1000 - <1500 | 13 | 135 | 0.69 (0.37-1.31) |
| ≥1500 | 190 | 1,822 | 0.94 (0.82-1.07) |
| *OR per 500 DDDs* | 114 | 1,127 | 0.89 (0.71-1.13) |
| *p-value* |  |  | *0.32* |

Abbreviations: CI, confidence interval; DDD, defined daily dose; OR, Odds ratio.

^1^ Adjusted for age, calendar time (by risk-set matching and the conditional analysis), chronic obstructive pulmonary disease, asthma, rheumatoid arthritis, polymyalgia rheumatica/ giant cell arthritis, psoriatic arthritis, ankylosing spondylitis, Crohn’s disease, ulcerative colitis, renal diseases, multiple sclerosis, and adrenal insufficiency, charlson comorbidity index score, educational, immunosuppressants, non-steroidal anti-inflammatory drugs, proton pump inhibitors, statins, low-dose aspirin and selective serotonin reuptake inhibitors.

**Appendix S1 – Danish Nationwide Health Registries**

The **Danish Cancer Registry** has recorded incident cases of cancer on a nationwide basis since 1943 and provides accurate and almost complete records of cancer cases in Denmark. Cancer diagnoses are coded according to the *International Classification of Diseases*, *Tenth Revision* 10 (ICD-10) and the ICD for Oncology (ICD-O-1-3) for topography and morphology (18).

The **Danish Pathology Register** contains records of pathological specimens, which for some departments dates back as early as 1970 (22). Since 1990, all departments of pathology have used electronic registrations and from 1997, it became a legal obligation to report the pathologies to the Danish Pathology Register. The registry contains coded diagnoses based on the Danish Systematized Nomenclature of Medicine (SNOMED).

The **Danish** **National Prescription Registry** contains data on all prescription drugs filled by Danish residents since 1995. The data include the type of drug, date of filling, and quantity (19). The dosing information and the indication for prescribing are not available and no information is available on drugs used at hospital level. Drugs are categorized according to the Anatomic Therapeutic Chemical (ATC) index, a hierarchical classification system developed by the World Health Organization, and the quantity dispensed for each prescription is described by the number and strength of the pharmaceutical entities (*e.g*., tablets), as well as defined daily doses (DDD).

The **Danish** **National Patient Registry** contains nationwide data on all non-psychiatric hospital admissions since 1977 and on ambulatory hospital contacts and psychiatric admissions since 1995 (20). Discharge/contact diagnoses have been coded according to ICD-8 from 1977 to 1993 and ICD-10 since 1994.

Statistics Denmark is a governmental institution that collects and processes information for a variety of statistical and scientific purposes, e.g. education and income. It hosts the **Population Education Registry,** which contains information on nearly all adult Danes and provides the highest completed level of education, defined as the longest duration of schooling (21).

The Danish **Civil Registration System** contains data on addresses, migration, and date of death (23,24). This system allowed us to extract population controls and to keep track of all subjects during the study period.

**Table S3– Codes and definitions**

| **Prostate cancer diagnosis and stage** | | |
| --- | --- | --- |
| **Prostate cancer cases**  Adenocarcinoma | *ICD-10*  *ICD–O–3* | C61.9  8140/3 |
| **Stage** |  |  |
| Localized | *TNM* | T1–4,x; N0; M0  T1–2; N0; Mx  T1–2; Nx; M0,x |
| Non localized | *TNM* | T1–4,x; N1; M0  T1–4,x; N0; M1  T1–4,x; N1; M1  T1–4,x; Nx; M1 |
| Others | *TNM* | T3–4,x; Nx; M0,x |
|  |  | T3–4,x; N0, Mx |
| **Exclusion criteria** | |  |
| Any cancer (except non-melanoma skin cancer) | *ICD-10* | C00-97 (except C44) |
| **Systemic glucocorticoids** | | H02AB |
| [Betamethasone](https://www.whocc.no/atc_ddd_index/?code=H02AB01&showdescription=yes) | *ATC code* | H02AB01 |
| [Methylprednisolone](https://www.whocc.no/atc_ddd_index/?code=H02AB04&showdescription=yes) | *ATC code* | H02AB04 |
| [Prednisolone](https://www.whocc.no/atc_ddd_index/?code=H02AB06&showdescription=yes) | *ATC code* | H02AB06 |
| [Prednisone](https://www.whocc.no/atc_ddd_index/?code=H02AB07&showdescription=yes) | *ATC code* | H02AB07 |
| [Hydrocortisone](https://www.whocc.no/atc_ddd_index/?code=H02AB09&showdescription=yes) | *ATC code* | H02AB09 |
| Other ([Triamcinolone, Dexamethasone)](https://www.whocc.no/atc_ddd_index/?code=H02AB08&showdescription=yes) | *ATC code* | H02AB08, H02AB02 |
| **Other drugs** |  |  |
| Immunossupressants | *ATC code* | L04 |
| Nonsteroidal anti-inflammatory drugs | *ATC code* | M01A |
| Proton pump inhibitors | *ATC code* | A02BC02 |
| Statins  Low-dose aspirin | *ATC code*  *ATC code* | C10AA  B01AC06, B01AC30, B01AC56, N02BA01 (≤100 mg), and N02BA51 (≤150 mg) |
| Selective serotonin reuptake inhibitors | *ATC code* | N06AB |
| **Prior diagnoses (diagnostic code or drug marker)** | | |
| Asthma | ICD-8  ICD-10 | 493  J45, J46 |
| Chronic obstructive pulmonary disease | ICD-8  ICD-10  ATC code | 491,492  J41–J44  R03BB, R03AC |
| Rheumatoid arthritis | ICD-8 | 712.0 712.1, 712.2, 712.3 712.5 |
|  | ICD-10 | M05-M06 |
| Polymyalgia rheumatica/ Giant cell arthritis | ICD-8  ICD-10 | 446.30, 446.31, 446.39  M315, M316, M35.3 |
| Psoriatic arthritis | ICD-8  ICD-10 | 696.09  M07.0-M07.3 |
| Ankylosing spondylitis | ICD-8  ICD-10 | 712.4  M45 |
| Crohn’s disease | ICD-8  ICD-10 | 563.01, 563.02, 563.09  K50 |
| Ulcerative colitis | ICD-8  ICD-10 | 563.1  K51 |
| Renal diseases | ICD-8  ICD-10 | 249.02, 250.02, 403, 404, 580-584, 590.09, 593.20, 753.10-753.19  N00, N01, N03, N04, N05 N06, N07, N08, N11, N14, N15, N16, N17, N18, N19, N26, N27, N28, N29, I12. I13, I15.0, I15.1, E10.2, E11.2, E14.2, Q61.1-Q61.4 |
| Multiple sclerosis | ICD-8  ICD-10 | 340  G35 |
| Adrenal insufficiency | ICD-8  ICD-10 | 253, 255.10, 255.11  E23.0, E24.0, E27.1, E27.2, E27.4, E89.3 |
| **Educational level** |  |  |
| Basic | *Duration* | 7-10 years |
| Medium | *Duration* | 11–12 years |
| Higher | *Duration* | ≥13 years |
| Unknown | *Duration* | - |
| NOTES:  ICD = International Classification of Disease  ATC = Anatomical Therapeutic Chemical  NCSP = Nordic Classification of Surgical procedures | | |
